# Antibody Testing and Lyme Disease Risk

**DOI:** 10.3201/eid1105.040381

**Published:** 2005-05

**Authors:** Elizabeth G. Stone, Eleanor H. Lacombe, Peter W. Rand

**Affiliations:** * Maine Medical Center Research Institute, South Portland, Maine, USA

**Keywords:** Borrelia burgdorferi, Lyme disease, canine

## Abstract

Lyme disease test results for >9,000 dogs were collected from participating veterinary clinics. Testing was conducted by using the IDEXX 3Dx kit, used widely by Maine veterinarians to screen clinically normal dogs during heartworm season. This study demonstrates how this test can be a valuable public health disease surveillance tool.

Lyme disease is the most commonly reported vectorborne disease in the United States; however, many experts believe that the number of cases is underreported. Lyme disease is often regarded as a routine condition or is frequently managed in high-volume settings (1). Few studies have assessed the accuracy of passive Lyme disease surveillance systems, but 1 study showed a 34% reporting rate (1). When tick identification services are offered, the identification data can show where disease vectors are found. In 1989, to determine the extent of the recently recognized infestation with *Ixodes scapularis*, the Maine Medical Center Research Institute's Vector-borne Disease Laboratory offered free tick identification to physicians, hospitals, veterinarians, and the general public. Since that time, >20,000 ticks, representing 14 species, have been identified. Testing has documented *Borrelia burgorferi* infection in *I. scapularis* from all Maine counties except 3.

Mapping of ticks submitted for identification is subject to certain biases, which limits its utility for predicting human risk. Submission rates vary depending on population, education, and local concern, and results show little about disease transmission, particularly in disease-emergent areas where infection rates may lag behind tick distribution. The limitations of passive Lyme disease surveillance and tick identification that provide geographic information about risk can be largely overcome by using canine seroprevalence studies. Dogs are sensitive indicators because they have greater exposure to ticks. In disease-endemic areas, ≥50% of unvaccinated dogs have been reported to be infected (2,3). The prevalence of Lyme borreliosis in dogs correlates with infection in humans (4,5), as well as entomologic indicators of disease transmission (6). A newly available enzyme-linked immunosorbent assay (ELISA) kit (SNAP 3Dx, IDEXX Laboratories, Westbrook, ME, USA) is used widely by veterinarians in Maine to screen dogs for *B. burgdorferi* and heartworm infection. This test is used as part of a health screen during the heartworm testing season and can potentially generate large volumes of unbiased test data for public health application.

The test kit detects antibodies directed against an invariable region (IR_6_) of the *B. burgdorferi* surface protein VlsE (Vmp-like sequence, Expressed) (2).The C_6_ ELISA test is not cross-reactive with antibodies induced by vaccination with either recombinant *B. burdorferi* outer-surface protein A (OspA) or whole-cell bacterin (2). This test has a very high accuracy rate, with 94.4% sensitivity and 99.6% specificity reported (7). In a clinical setting, when 18 dogs with known vaccination history were tested, the test results were 100% consistent with Western blot results (8).

## The Study

One hundred sixty-four Maine clinics were contacted in February 2003 and invited to join the study; 69 of these agreed to participate. Clinics were instructed to record results of all IDEXX 3Dx Lyme disease tests that were conducted as part of a routine health screen, to record town of residence, and to record if a Lyme disease vaccine had ever been administered. Lyme disease vaccines can be highly effective (2); however, since vaccination rates are unevenly distributed, inclusion of vaccinated dogs would bias estimates of disease risk. This protocol was approved by the Maine Bureau of Health Institutional Review Board.

Canine seroprevalence rates were calculated for minor civil divisions, including towns and unorganized townships. Rates were calculated only for divisions that had results of 10 or more tests. The relationships between the canine prevalence rates and human Lyme disease reports to the Bureau of Health (217 division-matched reports) and tick submissions to the Vector-borne Disease Laboratory (12,482 division-matched submissions) for the 2 years before this study, 2001–2002, were tested with Spearman rank correlation. Canine C_6_ antibodies persisted in experimentally infected, untreated dogs for ≥65 weeks, with no endpoint described (9); exposure status of the dogs in the present study could not be determined. Using data from 2 years allowed us to include sufficient numbers of human reports for meaningful statistic testing without sacrificing the ability to look at a "snapshot in time" of the Lyme disease spread.

**Figure 1:**
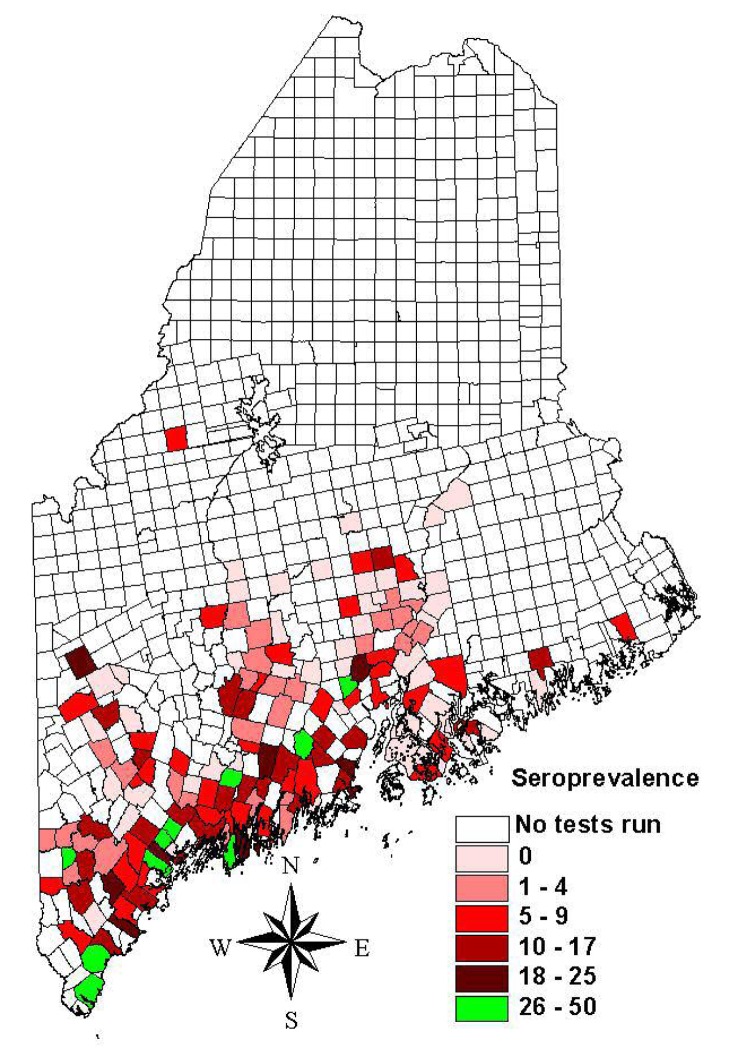
Canine Lyme disease seroprevalence rates based on the IDEXX 3Dx test for minor civil divisions with ≥10 tests, Maine, 2003.

**Figure 2:**
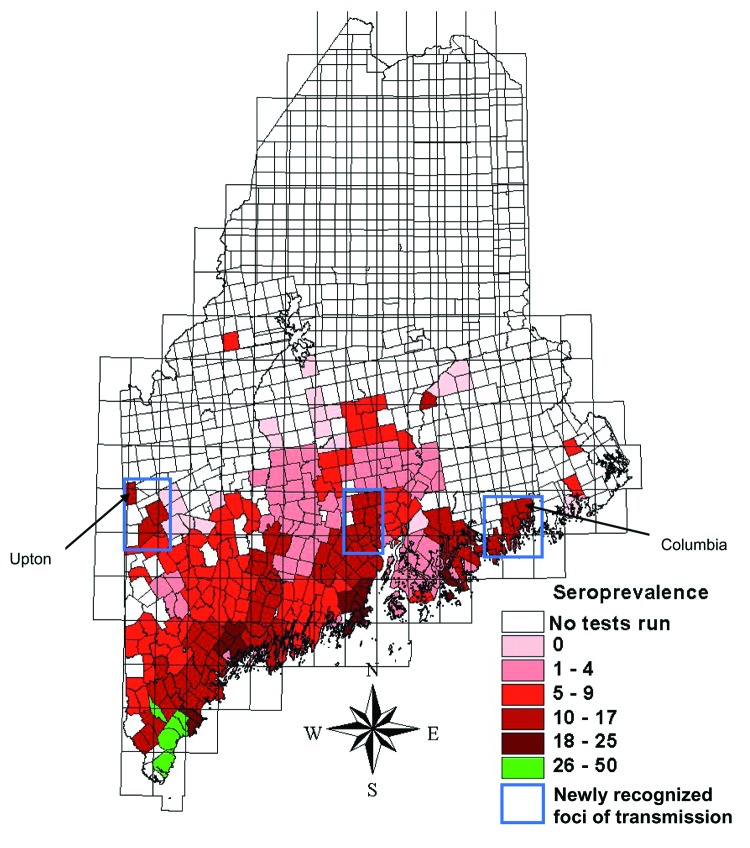
Regional canine Lyme disease seroprevalence rates calculated from minor civil division pools created within 15-minute quadrangles, Maine, 2003.

Two maps were created. The first map (Figure 1) showed prevalence rates of minor civil divisions with ≥10 tests. The second map (Figure 2) showed pooled data from all divisions, including those with small sample sizes. For this map, an overlay of the state with 15-minute quadrangles was used. Each division from which data were collected was assigned to the quadrangle that contained the largest portion of its area. Seroprevalence rates for quadrangles were calculated by combining test results from all divisions within a quadrangle to find the average rate. Divisions were then assigned the average seroprevalence rate of their quadrangle for mapping. Quadrangles having a pooled total of <10 tests were not included in this map.

Test results from 9,511 dogs that had not been vaccinated for Lyme disease were submitted from 343 minor civil divisions. Tests were performed from March to July 2003. The overall seroprevalence rate was 8%. One hundred and eighty-three divisions met the criterion of a minimum sample size of 10 for calculating prevalence rates. At the division level, seroprevalence rates significantly correlated with the number of ticks submitted to the Maine Medical Center Research Institute's Vector-borne Disease Laboratory from 2001 to 2002 (r = 0.41, p<0.001), and human Lyme disease reports to the Bureau of Health (r = 0.15, p<0.05) from 2001 to 2002.

Regional seroprevalence rates were calculated for 65 quadrangles representing 297 minor civil divisions. Seroprevalence rates ranged from 0% to 47%. Rates were highest along southern coastal Maine (≤47%), with regional rates of 11% as far east as Columbia and along the mid-New Hampshire border as far north as Upton. Forty-four divisions with ≥10 tests had prevalence rates of 0%; 12 of these had ≥30 tests and 3 had ≥60.

## Conclusions

This study demonstrates how canine serosurveys using the IDEXX 3Dx test can serve as an active surveillance system for potential human Lyme disease risk. This method overcomes the limitations of human Lyme disease reporting systems by relying on routine screening of populations of healthy dogs to calculate true seroprevalence rates. In this study, a large volume of data from across the state was generated for the most extensive and detailed measure of regional Lyme disease risk in Maine to date. In contrast, passive human Lyme disease surveillance during the previous 2 years yields cases from <90 towns, approximately two thirds of which had only 1 or 2 cases.

Canine seroprevalence rates were congruent with *I. scapularis* submissions and human Lyme disease reports during a 2-year period when dogs could have been infected, reinforcing the effectiveness of this method for predicting geographic human risk. One previous study has calculated canine seroprevalence rates in Maine (6), but a different assay technique was used (4), which limited our ability to compare those rates to those of the current study. In spite of substantial agreement between canine seroprevalence and rates of tick submissions, mapping of canine seroprevalence data shows high-risk foci in inland areas that were not previously identified by 14 years of tick submissions to the Vector-borne Disease Laboratory or from human Lyme disease reporting to the Bureau of Health; this suggests that canine serosurveys may identify new areas of disease transmission. These are areas of low human population density, and repeat surveys may demonstrate the value of canine serosurveillance in detecting disease spread where human populations are low.

Mapping of pooled data on a regional scale allows geographic patterns of disease to be viewed. Most notably, our data show a concentration of infected dogs in southern and coastal areas. Patterns of infection are suggested in inland areas as well. The significance of these patterns with respect to environmental variables favoring disease transmission is unknown but could be clarified by comparing prevalence rates with patterns of land use, deer herd density, habitat, and other ecologic attributes.

The widespread acceptance of the IDEXX 3Dx test facilitates the use of canine serosurveys for public health. In many Maine veterinary offices, virtually every dog tested for heartworm in the spring is tested for *B. burgdorferi* antibody; however, well below 100% of canine patients are vaccinated against Lyme disease. Test results can be collected opportunistically from collaborating veterinarians with minimal effort. Previous serosurveys have involved much more intensive effort because of the need for veterinarians to collect extra blood samples. The ease of data collection based on this manner of testing enhances real-time as well as long-term monitoring of disease. Furthermore, the large volumes of test results generated from routine *B. burgdorferi* screening, and the ability to collect information on dog residence, make large-scale studies of disease geography possible. That we did not exclude in our analyses dogs that have traveled suggests that caution should be used when considering the importance of low prevalence rates or prevalence rates calculated from low sample sizes. However, our finding of dozens of towns with 0% prevalence suggests that the effect of dogs that have traveled on calculated seroprevalence rates is small.
